# Reply to Morris et al. re: ‘The medical evidence on non-therapeutic circumcision of infants and boys—setting the record straight’

**DOI:** 10.1038/s41443-022-00631-y

**Published:** 2022-10-19

**Authors:** Matthew Deacon, Gordon Muir

**Affiliations:** Urology Department, King’s College Hospital, London, UK

**Keywords:** Urogenital diseases, Disease prevention

We read with interest the response by Morris et al. [1] to our recently published review, and thank them for their comment. Despite our efforts to provide a reasoned and balanced assessment of current evidence [2], they continue to rely heavily on self-cited and previously discredited studies, and repeatedly make inaccurate assessments of the quality of available evidence, based on entrenched and partisan opinion. Morris et al. refer to evidence of ‘numerous critiques’ by citing Morris’s own review paper which, as has been noted by others [3], includes over 70 citations of self-authored articles, made up significantly of ‘critical comments’ such as the one we respond to here.

Morris et al. start by referencing an article by El Bcheraoui et al. [4] on circumcision related complications. As already noted by others [5], one of the most significant flaws of this study, is in its short observation period of stricture formation at just 180 days post circumcision. This is far too short to be clinically relevant, as stricture formation can take months or years to become evident [6]. This renders the quoted complication rate of 0.4% inherently inaccurate, and far removed from the conclusions of other high-quality studies on the subject (estimating a rate of 7.29%) [7]. Even without this limitation, the main conclusion of the article relates to neonatal circumcision having a lower complication rate than circumcision of older children, and, as there is no separate analysis for complications in adult men, cannot be used to assess the advantages and disadvantages of waiting until a man is of an age to make the decision independently.

Morris et al. have again chosen to present their astounding claim that the benefits of circumcision exceed the risks by a ratio of ‘200:1’, an assertion that has not been repeated or replicated by any other scientist or recognised medical body [5]. This figure has been extensively debunked, including in our own review [2], because of a massive over-estimation of the protective effects of non-therapeutic circumcision (NTC) with a failure to present any less invasive options. Non-partisan organisations such as the Canadian Paediatric Society (CPS) have reaffirmed their position in not recommending routine NTC, stating that ‘the medical risk:benefit ratio of routine newborn male circumcision is closely balanced when current research is reviewed’ [8].

Regarding phimosis and normal development of the foreskin, Morris et al. criticise the articles included in our review relating to the prevalence of full retractability of the foreskin by age sixteen. They point instead to analysis by Yang et al. [9] in which the lower limit of the oldest age group is just eleven years old. It is misleading to use this as a comparator, as the penile development occurring between 11 and 16 is clearly very significant. Furthermore, Yang provides no reference to who was performing the examinations, or for what reason patients presented to hospital.

As discussed in our review [2], when assessing the effect of circumcision on incidence of urinary tract infection (UTI) Morris et al. [10] give a woefully inaccurate estimation of the lifetime incidence of UTI in uncircumcised males. The calculations they present are based on a tiny handful of adult men in a single study. They then proceed in trying to compare the number needed to treat (NNT) of an irreversible surgical procedure with that of a childhood vaccination programme, which is confused at best and dangerous at worse. There is already considerable mistrust among the lay public of even highly effective vaccines about which there is a medical consensus; the continued appeal to this analogy by Morris et al., especially in the face of conflicting evidence, threatens to damage pubic trust even more.

Morris et al. reference Eiselberg’s paper, citing a NNT of 39 circumcisions required to prevent one UTI, but the data in this paper concerns just 39 UTIs in almost 4000 person years of follow up. Morris et al. later then accept a NNT for UTI of 100, but get lost in a statistical debate regarding the total number of antibiotic courses which may result from ubiquitous uptake of infant NTC. The group fail to recognise that this tiny reduction in the number of children (in their example *n* = 10) taking a short course of antibiotics, is being compared against putting 1000 children through an irreversible surgical procedure. In other words, they are suggesting that a thousand men should have their ability to decide about the state of their genitalia removed, in order that a handful of children might be spared a seven-day course of antibiotics.

When discussing the effect of circumcision on incidence of STIs, Morris et al. again fail to present evidence which demonstrates an advantage of circumcision in preventing HIV transmission in a western population. His advocacy for universal infant circumcision as a prevention tool for HIV transmission in later life has now found to be unacceptable even to the United States President’s Emergency Plan for AIDS Relief (PEPFAR) programme, who dropped it as a recommendation owing to unacceptably high levels of injury [11].

Regarding penile cancer, Morris et al. acknowledge a NNT of around 1000 in the UK for neonatal circumcision, meaning 1000 children would be put through an operation to prevent a single case of penile cancer. Ethics aside, this is not a justifiable public health argument, particularly when considering the relatively good prognosis found in often easily diagnosed early-stage disease [12]. Morris et al. refer to an effect of circumcision on prostate cancer without reference to evidence supporting this claim. Regarding cervical cancer risk and circumcision, Morris et al. report that randomised control trial (RCT) data exists but fail to reference or quote it.

Concerning procedural and post-operative pain from infant circumcision, Morris et al. point to a claim by the CDC which has little evidence to back it up, as highlighted in our review. In the Freeman et al. paper that Morris et al. take exception to, 70.7% of parents reported at least some degree of pain when asked to rate ‘how much pain your child suffered from the circumcision’ [13], which is clearly far different from the ‘93.3%’ painless circumcision rate quoted in the CDC report.

As outlined in our review, the effect of circumcision on sexual function is difficult to quantify, but our balanced assessment of the evidence concluded that for this exact reason, the decision to circumcise should be left solely with the individual whose foreskin is being considered for removal. Some statements within this section of the debate can be taken as fact however, namely that any sensation felt within the foreskin itself is inherently removed following circumcision. Further to this, as it has been shown that the foreskin is the most sensitive part of the penis to light touch and mild warmth sensation for non-circumcised men [14, 15], it is hard to understand how it could not be involved in sexual function and experience, and thus a delayed decision for this reason seems both logical as well as ethical.

Finally, Morris et al. produce a table which attempts to compare the merits of neonatal circumcision with circumcision of ‘older boys and men’ [1]. The inclusion of older boys in this comparison fails to reflect one of the key conclusions of our review, and also omits the very reasonable option of not getting circumcised whatsoever. Whether something is ‘simple’ or ‘convenient’, whether the ‘cost is lower’, and whether it is without ‘anxiety’ or ‘embarrassment’, leads to the obvious question of ‘for whom?’. These considerations fail to hold the best interests of the child in mind, and do not seem adequate enough justification when explaining to an adult why this irreversible surgical procedure was performed on them as a child without their consent or consultation.

There are entrenched opinions here, both professional and socio-religious, which we fail to understand as open-minded surgeons. Unlike Morris et al., we do carry out hundreds of circumcisions each year, but these are on consenting patients for demonstrable benefits: we are not “anti-circumcision”. We fail to see how a medical argument can be made for non-therapeutic circumcision in those unable to give consent in western healthcare systems.
